# Transcription factor 7-like 2 (*TCF7L2*) gene polymorphism and clinical phenotype in end-stage renal disease patients

**DOI:** 10.1007/s11033-014-3275-6

**Published:** 2014-02-27

**Authors:** Monika Buraczynska, Pawel Zukowski, Piotr Ksiazek, Agata Kuczmaszewska, Joanna Janicka, Wojciech Zaluska

**Affiliations:** 1 Laboratory for DNA Analysis and Molecular Diagnostics, Department of Nephrology, Medical University of Lublin, Dr K. Jaczewskiego 8, 20-954 Lublin, Poland; 2 Department of Public Health, Medical University of Lublin, Lublin, Poland

**Keywords:** Cardiovascular disease, Diabetic nephropathy, End-stage renal disease, Risk allele, Single nucleotide polymorphism

## Abstract

Variants of the transcription factor 7-like 2 gene (*TCF7L2*) have been associated with type 2 diabetes and cardiovascular disease in different populations. Here we investigated the potential association of the rs7903146 polymorphism in the *TCF7L2* gene with clinical profile of end-stage renal disease (ESRD) patients. We examined a cohort of 1065 ESRD patients with diabetic and non-diabetic renal disease. The control group consisted of 924 healthy individuals. All subjects were genotyped for the rs7903146 single nucleotide polymorphism by polymerase chain reaction. The genotype distribution and allele frequencies were significantly different between ESRD patients and controls (*p* < 0.01). The OR for the TT genotype was 2.81 (95 % CI 2.08–3.79). Genotype and allele frequencies were compared between subgroups of patients with different clinical phenotypes. The frequency of the T allele was significantly higher in patients with diabetic nephropathy versus non-diabetic renal disease (*p* = 0.007, OR 1.70, 95 % CI 1.36–2.11). The statistically significant differences were demonstrated between patients with and without cardiovascular disease, with the OR for T allele 1.57 (95 % CI 1.31–1.90). The odds ratio for TT genotype was 2.38 (95 % CI 1.62–3.51). In our study the T allele of the rs7903146 SNP in the *TCF7L2* gene confers the risk of developing diabetic nephropathy. We described for the first time a strong relationship between the TCF7L2 gene variant rs7903146 and cardiovascular disease in end-stage renal disease patients.

## Introduction

Chronic kidney disease (CKD) is a major public health problem due to high prevalence (about 10 % of general population), reduction in life expectancy and quality of life of patients and enormous cost [1].

A combination of multiple genetic and environmental factors contributes to the pathogenesis of CKD leading to end stage renal disease (ESRD) [2–4]. The identification of causative genes predisposing to chronic renal disease and its complications could provide means to better understanding the pathogenesis of the disease and result in better prevention, diagnosis and treatment.

Majority of ESRD patients have comorbidities such as diabetes, hypertension and cardiovascular disease [5]. The prevalence of cardiovascular disease (CVD) is much greater in patients with chronic renal failure than in general population [6, 7]. The high prevalence of CVD and increased mortality rate in this patient population can be only in part explained by traditional risk factors such as hypertension, diabetes mellitus, hyperlipidemia and age that are common in dialyzed patients with ESRD [8, 9]. With the advent of genome-wide association scans, numerous risk variants have been identified as candidates for conferring susceptibility to renal and cardiovascular diseases but most of them with only modest effects [3, 10, 11].

Transcription factor 7-like 2 (TCF7L2) belongs to a family of TCF/lymphoid enhancer factor (LEF) transcription factors and is a key component of the Wnt signaling pathway involved in the regulation of pancreatic beta-cell proliferation, differentiation and insulin secretion [12]. It is involved in vascular remodeling through the regulation of smooth muscle cell proliferation and endothelial cell growth [13, 14]. TCF7L2 may therefore contribute to cardiovascular disease.

The gene encoding *TCF7L2* spans a 215,863 bases region on chromosome 10q25.3. It was first identified as a diabetes risk conferring gene in 2006 [15]. The most significant genetic association with diabetes was detected for two intronic single nucleotide polymorphisms (SNPs), rs7903146 (intron 3) and rs12255372 (intron 4) located 50 kb from each other [15–19]. These polymorphisms were also found to be linked to diabetic coronary atherosclerosis [20]. In a large, population-based study, the authors examined whether variants of TCF7L2 gene were associated with progression of chronic kidney disease and measures of kidney function. The results suggested that studied variants were significantly associated with reduced kidney function and CKD progression [21].

The aim of our study was to investigate the potential effect of the rs7903146 (C/T) polymorphism in intron 3 of the *TCF7L2* gene on clinical phenotype of end-stage renal disease. We have performed a case–control association study in a cohort of 1065 ESRD patients and 924 healthy controls.

## Subjects and methods

### Study subjects

The study population consisted of 1,065 unrelated, consecutive adult patients on maintenance dialysis. All subjects were Caucasians of Polish origin. Seventy two patients were treated with peritoneal dialysis and 993 were on hemodialysis. ESRD resulted from chronic glomerulonephritis (*n* = 332), diabetic nephropathy (*n* = 232), interstitial nephritis (*n* = 141), polycystic kidney disease (*n* = 86), obstructive nephropathy (*n* = 39) and other causes (*n* = 370). The mean time on dialysis was 4.4 years (range 2–35 years). Cardiovascular disease was diagnosed and documented in 672 patients, as one or the combination of several pathological states: congestive heart failure, left ventricular hypertrophy, angina pectoris, ischemic heart disease, myocardial infarction, peripheral arterial disease, ischemic cerebral stroke or atheromatous lesions, all confirmed by appropriate biochemical, radiographic, echocardiographic and vascular diagnostic criteria. There was a substantial overlap between categories. At the beginning of the study 821 patients were hypertensive and receiving antihypertensive medications. Hypertension was defined as a systolic blood pressure >140 mmHg and diastolic blood pressure >90 mmHg and/or use of antihypertensive medication. Healthy control subjects (*n* = 924) were randomly recruited, mostly among hospital staff members and blood bank donors who underwent health examination. All individuals had normal ECG and no clinical signs of renal disease or CVD. Written informed consent for participating in genetic studies was obtained from all ESRD and control subjects. The study protocol was approved by an institutional ethics committee. The investigation conforms to the principles of the Declaration of Helsinki.

### Genotyping

Genomic DNA was extracted from peripheral blood leukocytes (obtained from EDTA anticoagulated blood) using a standard technique. All subjects were genotyped for the rs7903146 SNP by polymerase chain reaction–restriction fragment length polymorphism assay (PCR–RFLP). Amplified fragments were digested with Rsa I restriction endonuclease (Fermentas GmbH, St Leon-Rot, Germany). DNA fragments were visualized on 3 % agarose gels. The CC, CT and TT genotypes (30 random samples for each genotype) were confirmed by automated sequencing in CEQ 8000 Genetic Analysis System (Beckman Coulter, High Wycombe, England). Observed concordance between genotyping assays was 100 %.

### Statistical analysis

Statistical calculations were performed using SPSS 11.0 for Windows (SPSS, Inc., Chicago, IL, USA). For baseline characteristics the normally distributed continuous variables are presented as mean ± SD. The Hardy–Weinberg equilibrium was verified with the χ^2^ test. Genotype distribution and allele frequencies were compared between groups using a Pearson χ^2^ test of independence with 2 × 2 contingency and *z* statistics. For continuous variables the *t* test and ANOVA were used for statistical significance. Where appropriate, the odds ratios (OR) with corresponding 95 % confidence intervals (CI) were calculated for the effects of high-risk allele and for genotypes. Logistic regression analysis was performed for evaluating the association of the TCF7L2 variant with clinical phenotypes. A two-tailed type I error rate of 5 % was considered statistically significant. Power calculations were done using on-line available power calculator (http://calculators.stat.ucla.edu).

## Results

The genotype of the rs7903146 polymorphism in the *TCF7L2* gene was determined in 1065 ESRD patients and 924 healthy individuals. The details of clinical and biochemical characteristics of studied subjects are presented in Table 1.

**Table 1:** Demographic and clinical profile of studied subjects

Variable	ESRD patients	Controls	*p* value^a^
N	1065	924	
Male/female	611/454	492/432	
Age at study (years)	59.4 ± 18	51 ± 17	<0.001
Years on dialysis	4.8 ± 3.6	NA	
Diabetes mellitus (%)	198 (19)	0	
Hypertension (%)	768 (72.1)	0	
BMI (kg/m^2^)	27.2 ± 4.4	26.2 ± 3.4	<0.001
Serum creatinine (μmol/l)	792.3 ± 158	ND	
Total cholesterol (mmol/l)	5.2 ± 1.73	3.9 ± 1.71	<0.001
HDL cholesterol (mmol/l)	1.22 ± 0.81	ND	
Triglycerides (mmol/l)	2.3 ± 1.78	1.17 ± 0.93^b^	<0.001

Values are presented as mean ± SD or numbers (%)

*ESRD* end-stage renal disease, *NA* not applicable, *ND* not determined

^a^Where significant

^b^Triglycerides were determined in 23 % of control subjects

Genotype data were successfully obtained for all subjects. The genotype and allele frequencies in the control group were similar to those previously reported for other European populations [22, 23] and the genotype distribution did not deviate from Hardy–Weinberg equilibrium (*p* > 0.05). Table 2 shows the genotypes of rs7903146 SNP in end-stage renal disease patients and controls. The genotype distribution and allele frequencies were significantly different between the entire ESRD group and healthy individuals (*p* < 0.01). The odds ratio (OR) for the T allele (minor allele) was 1.65 (95 % CI 1.44–1.89) and for the TT genotype vs. CC 2.81 (95 % CI 2.08–3.79).

**Table 2:** Genotype and allele distribution of rs7903146 SNP in the TCF7L2 gene in ESRD patients and controls

Subjects	CC	CT	TT	MAF	*p* value	OR (95 % CI) for T allele	
						Unadjusted	Adjusted^a^
ESRD (*n* = 1065)	426 (40)	458 (43)	181 (17)	0.38	0.008	1.65 (1.44–1.89)	1.58 (0.87–2.84)
Controls (*n* = 924)	490 (53)	360 (39)	74 (8)	0.27		1.0 (ref.)	

Data are *n* (%). HWE test: X^2^ = 0.48, *p* = 0.488422. Statistical power for ESRD versus controls = 99.9 %

*ESRD* end stage renal disease, *MAF* minor allele frequency

^a^OR adjusted for age, gender, BMI and lipid profile

Genotype and allele frequencies were compared in subgroups of ESRD patients with different clinical phenotypes. The results are summarized in Table 3. The comparison of subgroups with diabetic and non-diabetic renal diseases showed the difference in genotype and allele distribution. The frequency of the T allele was significantly higher in patients with diabetic nephropathy than in those with non-diabetic renal disease (0.49 and 0.36, respectively, p = 0.007). The odds ratio for the T allele was 1.70 (95 % CI 1.36–2.11) and for the TT genotype was 2.61 (95 % CI 1.69–4.04). When only patients with non-diabetic renal disease were compared with controls there were also differences in allele and genotype distribution. The frequency of the T allele in patients and controls was 36 and 27 %, respectively (*p* = 0.04). The OR for the T allele was 1.50, CI 1.29–1.72.

**Table 3:** Genotype and allele distribution of rs7903146 SNP in the TCF7L2 gene in ESRD patients with different clinical phenotypes

Subjects	CC	CT	TT	MAF	OR (95 % CI) for T allele	
					Unadjusted	Adjusted^a,b^
ESRD (*n* = 1065)	426 (40)	458 (43)	181 (17)	0.38	–	–
ESRD CVD+ (*n* = 672)	238 (36)	298 (44)	136 (20)	0.42	1.57 (1.31–1.90)	1.52 (1.11–2.21)^a^
ESRD CVD− (*n* = 393)	188 (48)	160 (41)	45 (11)	0.32	1.0 (ref.)	
DN (*n* = 198)	53 (27)	96 (48)	49 (25)	0.49	1.70 (1.36–2.11)	1.78 (1.06–3.14)^b^
Non-diabetic (*n* = 867)	373 (43)	362 (42)	132 (15)	0.36	1.0 (ref.)	
Time to ESRD <10 years (*n* = 586)	226 (39)	252 (43)	108 (18)	0.40	1.14 (0.96–1.36)	–
Time to ESRD ≥10 years (*n* = 479)	200 (42)	206 (43)	73 (15)	0.37	1.0 (ref.)	

Data are n (%). Statistical power for DN versus non-diabetic = 95.7 %, for CVD+ versus CVD− = 94.8 %

*ESRD* end-stage renal disease, *CVD* cardiovascular disease, *MAF* minor allele frequency

^a^OR adjusted for age, gender, BMI, lipid profile, diabetes and hypertension

^b^OR adjusted for age, gender, BMI, lipid profile and hypertension

The statistically significant differences were demonstrated between subgroups of patients with cardiovascular disease and those without cardiovascular comorbidity. The frequency of the T allele was 0.42 versus 0.32 (*p* = 0.032) and OR was 1.57 (95 % CI 1.31–1.90). The odds ratio for TT genotype was 2.38 (95 % CI 1.62–3.51).

When diabetic subjects with and without CVD were compared with non-diabetic subjects with and without CVD, there were no statistically significant differences in observed associations. The statistical power for DM patients with CVD versus no CVD was 96.1 % and for non-diabetic patients with CVD versus those without it, the power was 95.4 %.

There was no significant difference in the genotype and allele distribution between patients with faster disease progression (time to ESRD <10 years) and those with slower progression (time to ESRD ≥10 years) (*p* = 0.534).

Multivariate logistic regression analysis was used to assess the role of the rs7903146 genotype and other coexisting factors in ESRD and CVD. These adjustments did not substantially affect the OR estimates. After confounding effects of potentially significant variables: age, gender, BMI, lipids, diabetes and hypertension were adjusted, the T allele and TT genotype were still significantly associated with ESRD and cardiovascular comorbidity in our study population.

## Discussion

So far *TCF7L2* is the most significant and reproducible susceptibility gene for type 2 diabetes in various ethnic groups [24–28]. In the present study we investigated the potential effect of the rs7903146 (C/T) polymorphism in intron 3 of the *TCF7L2* gene on clinical phenotype of diabetic and non-diabetic end-stage renal disease. This case–control association study involved 1,065 patients with end-stage renal disease and 924 healthy controls. The comparison of genotype and allele distribution between these two groups showed that the T allele of the rs7903146 polymorphism is significantly associated with ESRD. This is in agreement with previous findings of the association of *TCF7L2* genetic variants with renal function and progression of chronic kidney disease [21, 29]. *TCF7L2* as the diabetes-susceptibility gene may increase the risk of chronic kidney disease not only through its effect on diabetes but also through some renal-specific mechanisms. The product of the *TCF7L2* gene, TCF4, is a downstream effector in the Wnt signaling pathway, essential in the developing kidney [30]. It was also reported that mutations in the Wnt signaling pathway’s components are involved in inherited kidney diseases [31].

In our study genotype and allele frequencies were compared in subgroups of ESRD patients with different clinical phenotypes. The comparison of subgroups with diabetic and non-diabetic renal diseases showed the difference in genotype and allele distribution. The frequency of the T allele was significantly higher in patients with diabetic nephropathy than in those with non-diabetic renal disease. This result successfully replicated our previous observations that in type 2 diabetes patients the T allele is strongly associated with nephropathy, especially in patients with early onset of diabetes [32].

Diabetes and chronic kidney disease have an inflammatory response component in common and since the Wnt signaling pathway is involved in this response, studying a role of TCF7L2 gene variants in both diseases can improve the understanding of mechanisms involved in affecting disease risk.

In our study rs7903146 SNP was strongly associated with cardiovascular disease in ESRD patients. The T allele carriers had a higher risk of developing CVD. There were 65 % of carriers in the CVD+ subgroup of patients compared to 51 % in the CVD-subgroup. There were previous studies reporting the association of TCF7L2 variant rs7903146 with several diabetic micro- and macroangiopathic complications. Ciccacci et al. [33] found an association of this variant with diabetic retinopathy, cardiovascular disease and coronary artery disease in their group of 154 type 2 diabetes patients. Muendlein et al. [20] reported a strong correlation between the rs7903146 and coronary artery disease in his study of 393 type 2 diabetes patients. They hypothesized that TCF7L2 could directly influence the regulation of smooth muscle cell proliferation and the NF_KB pathway through Wnt signaling pathway. The effect of TCF7L2 rs7903146 variant was observed also in non-diabetic individuals [34]. It was found that the T allele of this SNP was associated with a higher prevalence and severity of coronary atherosclerosis in individuals without diabetes. The increased CVD risk associated with the rs7903146 SNP in our and other studies might be specific to diabetes and/or renal disease in studied patients. The atherosclerosis risk in communities study did not find significant association between TCF7L2 SNPs and incident vascular events [35].

As most of the association studies, ours has some limitations. Since it is a retrospective case–control study, a selection bias cannot be excluded. To limit this bias we included consecutive patients in the study and tried to adjust for known confounding risk factors. Our CVD subgroup of ESRD patients included atherosclerotic as well as non-atherosclerotic CVD phenotypes that could be related to renal failure or hypertension. Another limitation is that we did not perform any tests for silent cardiovascular disease in asymptomatic patients. The strength of our study is that all patients and controls are of the same ethnic origin. Furthermore, all subjects were examined in a standardized manner, with well defined diagnostic criteria and genotyping was performed blind with respect to case–control status. The sample size in our study had 99.9 % power to detect the genetic effect of *TCF7L2* allele at a p value of 0.05.

In conclusion, the findings of our study show that the T allele of the rs7903146 SNP in the *TCF7L2* gene confers the risk of developing diabetic nephropathy. We described for the first time a strong relationship between the TCF7L2 gene variant rs7903146 and cardiovascular disease in end-stage renal disease patients. Our findings might provide evidence showing that TCF7L2 plays an important role in the relationship between chronic kidney disease and CVD. Further independent studies are needed to replicate our observations and to elucidate the underlying mechanisms.
